# 

**Published:** 1884-11

**Authors:** 


					﻿NOVEMBER, 1884.
THIRTY-FIRST YEAR.
HALT’S
JOURNAL
OF
HEALTH
Guaranteed Circulation 200,000 Copies in 12 Months.
E. H. GIBBS, A.M., M.D., Editor.
No. 21 CLINTON PLACE EIGHTH STREET NEW YORK.
TERMS: $1 a Year.
Single number. 10 cts.
				

